# Efficacy and safety of ticagrelor monotherapy in patients following percutaneous coronary intervention: A systematic review and meta-analysis

**DOI:** 10.1097/MD.0000000000026070

**Published:** 2021-05-21

**Authors:** Wen-bin Zhang, Li-nan Liu, Yang Liu, Zhen Wang

**Affiliations:** a The First Clinical Medical School, Shandong University of Traditional Chinese Medicine; b Department of Cardiology, Affiliated Hospital of Shandong University of Traditional Chinese Medicine, Jinan, China.

**Keywords:** all cause death, bleeding, dual antiplatelet therapy, percutaneous coronary intervention, stent thrombosis, ticagrelor

## Abstract

**Background:**

We aimed to systematically evaluate the efficacy and safety ticagrelor monotherapy following percutaneous coronary intervention.

**Methods:**

Online databases were searched for relevant studies (published between the years 2015 and 2020) comparing 1-month Dual antiplatelet therapy (DAPT) followed by 23-month ticagrelor monotherapy with 12-month DAPT followed by 12-month aspirin monotherapy following percutaneous coronary intervention. Primary outcomes assessed efficacy whereas secondary outcomes assessed safety. Odds ratios (OR) with 95% confidence intervals (CIs) based on a random effect model were calculated and the analysis was carried out by the RevMan 5.3 software.

**Results:**

Only 6 studies were selected for this meta-analytical research. The meta-analysis results: MI(OR:0.96, 95% CI:0.86–1.06, *P* = .40), stroke (OR:1.04, 95% CI: 0.87–1.25, *P* = .68), stent thrombosis (OR: 0.91,95% CI:0.76–1.10,*P* = .32),New-Q Wave (OR:0.85,95% CI: 0.72–1.00, *P* = .05), all cause death (OR:0.91, 95% CI: 0.87–0.96, *P* < .0001), death from cardiovascular (OR: 0.76, 95% CI: 0.58–0.99, *P* = .04), revascularization (OR: 0.93, 95% CI: 0.87–0.99, *P* = .03). Ticagrelor monotherapy was associated with a significantly lower rate of myocardial Infarction (MI), stroke, stent thrombosis, all cause death, death from cardiovascular and revascularization (OR:0.91,95% CI:0.87–0.96, *P* < .0001) when compared to DAPT. Besides, DAPT was associated with a significantly higher rate of BARC3 or 5 bleeding (OR:0.85, 95% CI: 0.68–1.06; *P* = .16) when compared to ticagrelor. When bleeding was further subdivided, minor or major bleeding was also significantly higher with DAPT (OR: 0.72, 95% CI: 0.41–1.27; *P* = .26). GUSTO moderate or severe bleeding was also significantly higher with DAPT (OR: 0.77, 95% CI: 0.39–1.52; *P* = .45).

**Conclusion:**

Ticagrelor monotherapy after short-term dual-antiplatelet therapy (DAPT) can optimize ischemic and bleeding risks. And, it can reduce the occurrence of events outcome (MI, revascularization, stroke, stent thrombosis).

## 1 Introduction

Dual antiplatelet therapy (DAPT) with aspirin and ticagrelor is considered as the key element to prevent stent thrombosis following percutaneous coronary intervention (PCI) with drugeluting stents.^[1]^ In order to prevent long-term recurrent events and stent thrombosis in patients with Coronary atherosclerotic heart disease who are treated with drugeluting stents, aspirin and ticagrelor is usually recommended for at least 1 year.^[2]^ However, in recent years, as the risk of bleeding caused by double antiplatelet treatment has increased,^[3]^ more and more clinical reports have explored the efficacy and safety of ticagrelor monotherapy after 1 month of dual antiplatelet therapy after PCI.^[4–6]^

Ticagrelor is a platelet aggregation inhibitor. Its clinical efficacy and safety have been verified and supported by the platelet inhibition and patient outcome study (PLATO study) and its multiple subgroup studies.^[7]^ The PLATO study also shows that the efficacy of ticagrelor is significantly better than clopidogrel,^[8]^ so it has been listed in the first-line recommendation by many guidelines, and the ESC guidelines 2017 shown that in patients with acute coronary syndrome (ACS) when treatment with one of the more potent P2Y12 inhibitors, such as ticagrelor should be used instead of clopidogrel.^[9]^

As ACS continues to grow today, antiplatelet therapy is still one of the most important treatment measures for ACS. Ticagrelor is a new type of cyclopentyltriazole pyrimidine oral antiplatelet drug^[10]^. Ticagrelor is a nonprodrug, it can take effect directly without being activated by liver metabolism, and binds reversibly to the P2Y12 ADP receptor. The results of the PLATO study showed that ticagrelor treatment for 12 months without increasing major bleeding, compared with clopidogrel, further significantly reduced the risk of cardiovascular death/MI /stroke composite endpoint events in ACS patients by 16%, and at the same time significantly reduce cardiovascular deaths by 21%.^[11]^ Based on the benefits of ticagrelor treatment for ACS patients, relevant domestic and foreign guidelines recommend that ticagrelor be used for antiplatelet therapy for ACS patients. In the 2 authoritative guidelines of the European Cardiology Association, it is pointed out that clopidogrel can only be used in patients who cannot receive ticagrelor treatment, which is also sufficient Shows the acceptance of new drugs to further reduce mortality.^[12]^ So, we explored the efficacy and safety of ticagrelor monotherapy after 1 month of dual antiplatelet therapy after PCI.

In this analysis, we aimed to systematically compare the efficacy and safety between 1-month DAPT followed by 23-month ticagrelor monotherapy and 12-month DAPT followed by 12-month aspirin monotherapy, using a large number of patients which were extracted from recent 5-year publications (2015–2020).

## 2 Methods

### 2.1 Data sources and search strategy

PubMed, Embase, and the Cochrane library databases were searched for relevant publications (between the years 2015 and 2020) comparing 1-month DAPT followed by 23-month ticagrelor monotherapy with 12-month DAPT followed by 12-month aspirin monotherapy following PCI.

The following searched terms or phrases were used: “ticagrelor,” “percutaneous coronary intervention,” “”RCT.”

In addition, other terms also included in this study related to this particular topic, for example, “Brilique,” “AZD 6140,” “randomized controlled tria,” “Coronary Intervention, Percutaneous” et al.

### 2.2 Inclusion and exclusion criteria

Studies were included if:

1. They compared 1-month DAPT followed by 23-month ticagrelor monotherapy with 12-month DAPT followed by 12-month aspirin monotherapy following PCI.
2. They reported adverse clinical outcomes (assessing efficacy or safety) during 1 or 2 years follow-up period after PCI.
3. Study type: randomized controlled trial.

Studies were excluded if:

1. They did not compare efficacy and safety of ticagrelor monotherapy in patients following percutaneous coronary intervention, but instead, compared with ticagrelor plus aspirin vs aspirin monotherapy, or compared with other antiplatelet drugs with aspirin.
2. They did not report adverse outcomes which were associated with ticagrelor plus aspirin as their clinical endpoints.
3. Study type was not randomized controlled trial.
4. They outcome data which could be incomplete or unavailable.

### 2.3 Primary outcomes

Primary outcomes assessed efficacy included:

- All-cause death;
- Stroke;
- Myocardial infarction (MI);
- Stent thrombosis;
- NEW-Q Wave;
- Death from cardiovascular;
- Revascularization.

### 2.4 Secondary outcomes

Secondary outcomes which assessed safety included:

- -BARC 3 or 5 bleeding: The key secondary safety endpoint was site-reported bleeding assessed according to the Bleeding Academic Research Consortium (BARC) criteria(grade 3 or 5).^[13]^
- -TIMI (Thrombolysis in MI) major or minor bleeding^[14]^;
- GUSTO (Global Utilization of Streptokinase and Tissue Plasminogen Activator for Occluded Coronary Arteries) moderate or severe bleeding.^[15]^

In this analysis, the follow up time period was from 1 to 2 years following PCI and use of ticagrelor monotherapy.

The reported adverse clinical outcomes and the follow-up time periods have been listed in Table 1.

**Table 1:** Reported adverse clinical outcomes (efficacy and safety outcomes).

Author and year	Reported outcomes	Follow up periods
Pascal 2018	BARC 3 or 5 bleeding, New-Q Wave, MI, all-cause death stroke, stent thrombosis, revascularization	2 year
Mehran 2019	All cause death, BARC 3 or 5 bleeding, MI, stroke, stent thrombosis, death from cardiovascular	1 year
Franzone 2019	All cause death, BARC 3 or 5 bleeding, revascularization, stroke, MI, stent thrombosis, death from cardiovascular	2 year
Tomaniak 2019	All cause death, BARC 3 or 5 bleeding, New-Q Wave, Stroke, MI, stent thrombosis, revascularization	1 year
Leonardi 2019	BARC 3 or 5 bleeding, new-Q wave, all-cause death	2 year
Takahashi 2019	BARC 3 or 5 bleeding, new-Q wave, All-cause death, stroke, MI, stent thrombosis, revascularization	2 year

### 2.5 Data extraction

WZ, LL, YL, and ZW independently reviewed the data. The extracted information mainly includes:

1. the first author of the study and the publication time of the article;
2. the methodological quality evaluation elements;
3. the age, Gender, number of included cases, specific intervention measures, etc.;
4. Outcome indicators;
5. systematic extraction of patients participating in the study and other comorbid diseases.

**Figure 1:**
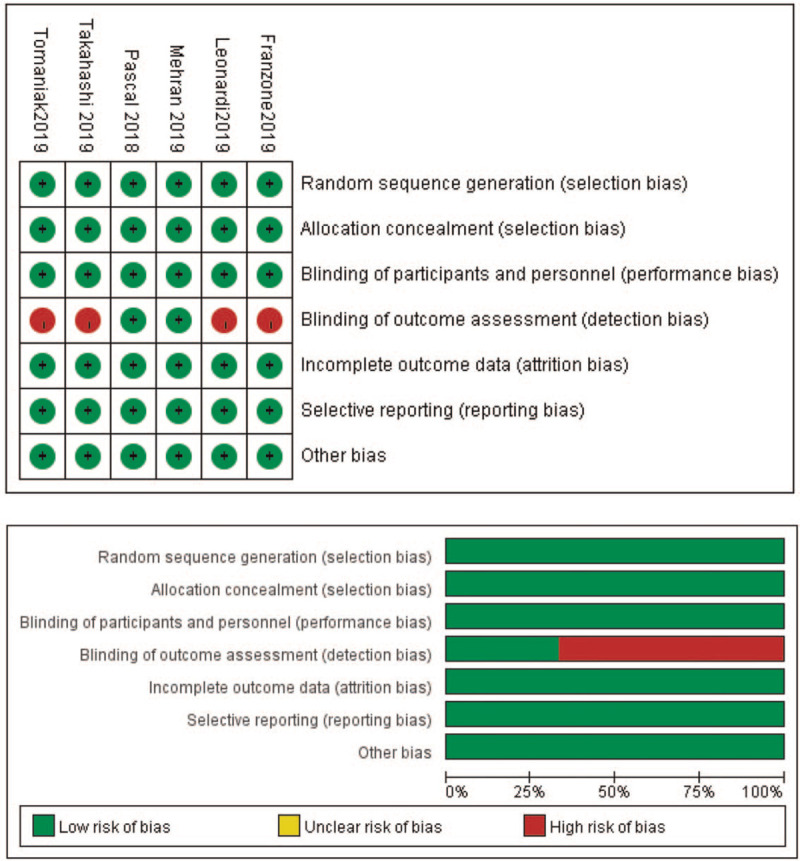
Risk of bias in included studies.

The quality evaluation is based on the Cochrane Collaboration, including selection bias, performance bias, detection bias, attrition bias, reporting bias, and other bias. In the statistical process, the quality assessment is classified: 5 or more are low risk of bias; 3 to 4 are moderate risk of bias; 3 or less are high risk of bias. Scores were given to each of the 7 components which were recommended by the Cochrane Collaboration (Fig. 1).

### 2.6 Assessment of heterogeneity reported bias and statistical analysis

The Preferred Reporting Items for Systematic Reviews and Meta-Analyses guideline was considered relevant for this meta-analysis.^[16]^

Heterogeneity which was an important feature in this analysis was assessed by 2 very basic statistical techniques: primarily by the Cochrane Q-statistic test (*P* < .05 was considered statistically significant; statistically supporting the drug which is being favored) and secondly by the *I*^2^-statistic test which was obtained following the subgroup analyses. A low value of *I*^2^ indicated a low heterogeneity whereas an increased heterogeneity was represented by a high *I*^2^ value.

When the heterogeneity test result of the included study *P* > .1 or *I*^2^≤50%, the Fixed-effects model is used for meta-analysis; when the heterogeneity test result *P* ≤ .1 or *I*^2^ > 50%, the Random-effects model is used for meta-analysis. We calculated odds ratios (OR) and 95% confidence intervals (CIs) which were generated through the RevMan 5.3 software.

Sensitivity analysis was be carried out when the heterogeneity test indicates significant heterogeneity among the included studies. Sensitivity analysis is used to evaluate the stability and reliability of the combined results of the meta-analysis, by assessing whether the combined results are affected by a single study and have significant changes. By excluding the documents one by one: check whether the heterogeneity has changed after culling one by one. If it is found that the heterogeneity has changed after excluding a certain study, then this article may be the source of the heterogeneity. After removing the article, we will again perform meta-analysis. If the included documents are removed, their heterogeneity remains unchanged, indicating that the results are relatively robust.

Publication bias which could possibly be present was estimated by observing funnel plots.

### 2.7 Ethics

Ethical committee or medical institutional board approval was not required for systematic reviews and meta-analyses.

## 3 Results

### 3.1 Searched outcomes

**Figure 2:**
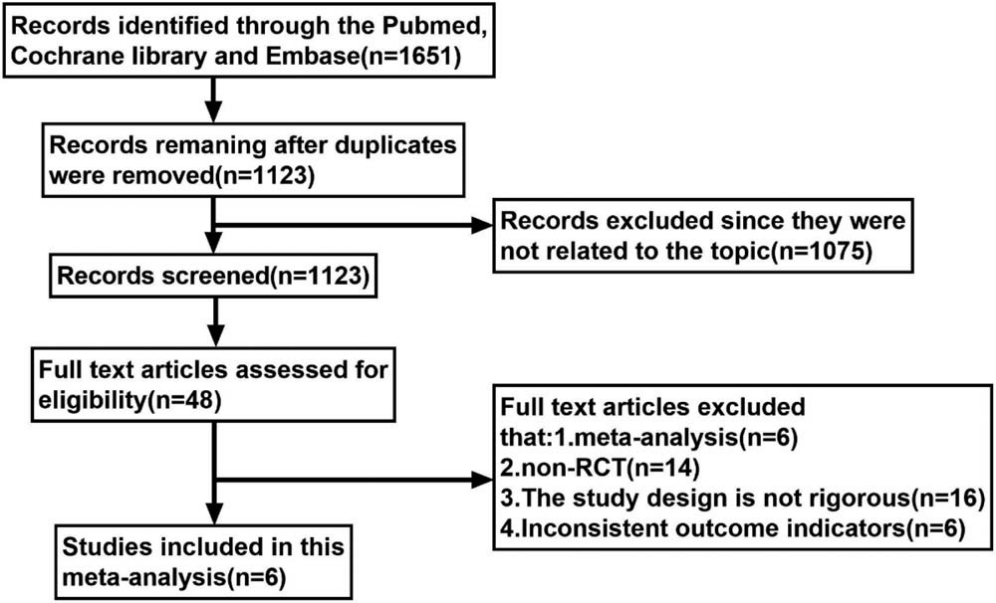
Flow chart of literature selection.

One thousand six hundred fifty one (1651) articles were obtained from the Pubmed, Cochrane Library, and Embase databases. One thousand seventy five (1075) articles were not related to this meta research and were therefore eliminated. Forty eight (48) full-text articles were finally reviewed for eligibility. Six article were eliminated since they were meta-analyses. Fourteen (14) articles were eliminated since they were non-RCT whereas another 16 articles were eliminated since their study design were not rigorous. At last, a further 6 articles were eliminated since they had inconsistent outcome indicators. Finally, only 6 studies^[17–22]^ were selected for this meta analytical research (Fig. 2).

### 3.2 Description of studies

Six studies with a total number of 57,703 patients (28,466 patients were treated with ticagrelor monotherapy and 29,237 patients were treated with DAPT) were included in this analysis.

A total number of 32,741 patients had ACS (17,264 patients were assigned to the ticagrelor monotherapy group and 15,477 were assigned to the DAPT group) including 5455 patients who had ST segment elevation myocardial infarction (STEMI) (3064 patients were classified in the ticagrelor monotherapy group vs 2391 patients which were classified in the DAPT group) and 11,229 patients who had non-ST segment elevation myocardial infarction (NSTEMI)(5590 patients were treated by ticagrelor monotherapy versus 5639 patients which were treated by DAPT),7970 patients who had Unstable angina (UA)(3972 patients were treated by ticagrelor monotherapy vs 3998 patients which were treated by DAPT. The remaining participants were patients suffering from stable coronary artery disease.

Patients were enrolled between the years 2013 and 2017. Patients from several corners around the globe countries. This current analysis consisted of studies which were published between the years 2015 to 2020. The main features of these studies have been summarized in Table 2.

**Table 2:** General features of the studies which were included in this analysis.

			ACS						
Author and year	No of patients in the DAPT group (n)	No of patients in the ticagrelor group (n)	STEMI Exp/Cont	NSTEMI Exp/Cont	UA Exp/Cont	Stable coronary artery Disease Exp/Cont	Year of patients’ enrollment	Countries of patients’ enrollment	Type of study
Pascal 2018	7988	7980	1062/1030	1684/1689	1004/1018	4230/4251	2013–2015	18 countries.	RCT
Mehran 2019	3564	3555	–	1024/1096	1249/1245	1047/999	2015–2017	11 countries.	RCT
Franzone 2019	3791	3794	689/665	760/737	490/499	1855/1890	2013–2015	11 countries.	RCT
Tomaniak 2019	3737	3750	1062/1030	1684/1689	1004/1018	–	2013–2015	-	RCT
Leonardi 2019	8383	7585	4638/2849	3745/4736	2013–2015	–	RCT		
Takahashi 2019	1774	1802	251/266	438/428	225/218	888/862	2013–2015	-	RCT
Total no of patients (n)	29237	28466	17264/15477	11765/12738					

### 3.3 Baseline characteristics

Baseline features of the patients have been summarized in Table 3. The patients had a mean age which varied between 64.5 and 65.4 years. The percentage of patients with other comorbidities has been summarized in Table 3.

**Table 3:** Baseline features of the studies which were included in this analysis.

Study	Age (years) Exp/Cont	Females (%) Exp/Cont	HTN (%)	DM (%)	Cs (%)	Pmi (%)	Pvd (%)
Pascal 2018	64.5/64.6	23.4/23.1	74.0/73.3	25.7/24.9	25.9/26.3	23.0/23.6	6.0/6.7
Mehran 2019	65.2/65.1	23.8/23.9	72.6/72.2	37.1/36.5	20.4/23/1	28.7/28.6	6.9/6.8
Franzone 2019	64.9/64.8	24.0/23.5	72.5/72.3	24.3/23.7	28.6/29.1	22.9/23.6	6.7/7.9
Tomaniak 2019	–	22.9/23.2	67.9/68.6	21.3/21.6	33.6/34.3	18.6/18.3	5.3/5.1
Leonardi 2019	64.9/64.2	23.7/22.8	72.6/74.5	24.0/26.5	28.8/23.7	23.3/23.3	7.3/5.5
Takahashi 2019	65.2/65.4	21.6/20.2	74.3/71.8	24.6/25.6	26.9/26.5	20.9/20.1	6.8/6.8

Overall, no significant difference in baseline features was observed between the 2 groups.

### 3.4 Primary outcomes (outcomes representing efficacy)

**Figure 3:**
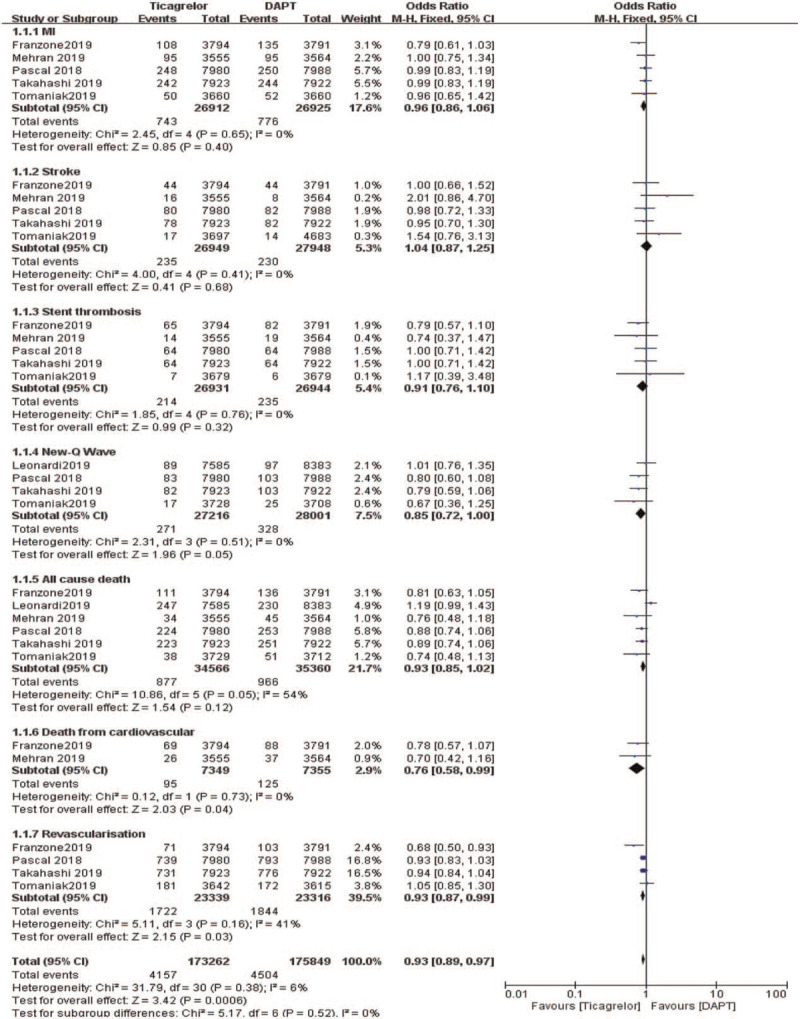
Comparing the efficacy (primary outcomes) observed between ticagrelor and DAPT.

Meta-analysis results: MI (OR:0.96, 95%CI: 0.86–1.06, *P* = .40), stroke (OR:1.04, 95%CI: 0.87–1.25, *P* = .68), stent thrombosis (OR:0.91, 95%CI: 0.76–1.10, *P* = .32), New-Q Wave (OR: 0.85, 95%CI: 0.72–1.00, *P* = .05), all cause death (OR: 0.93, 95%CI: 0.85–1.02, *P* = .12), death from cardiovascular (OR:0.76, 95%CI: 0.58–0.99, *P* = .04), revascularization (OR: 0.93, 95%CI: 0.87–0.99, *P* = .03; Fig. 3).

**Figure 4:**
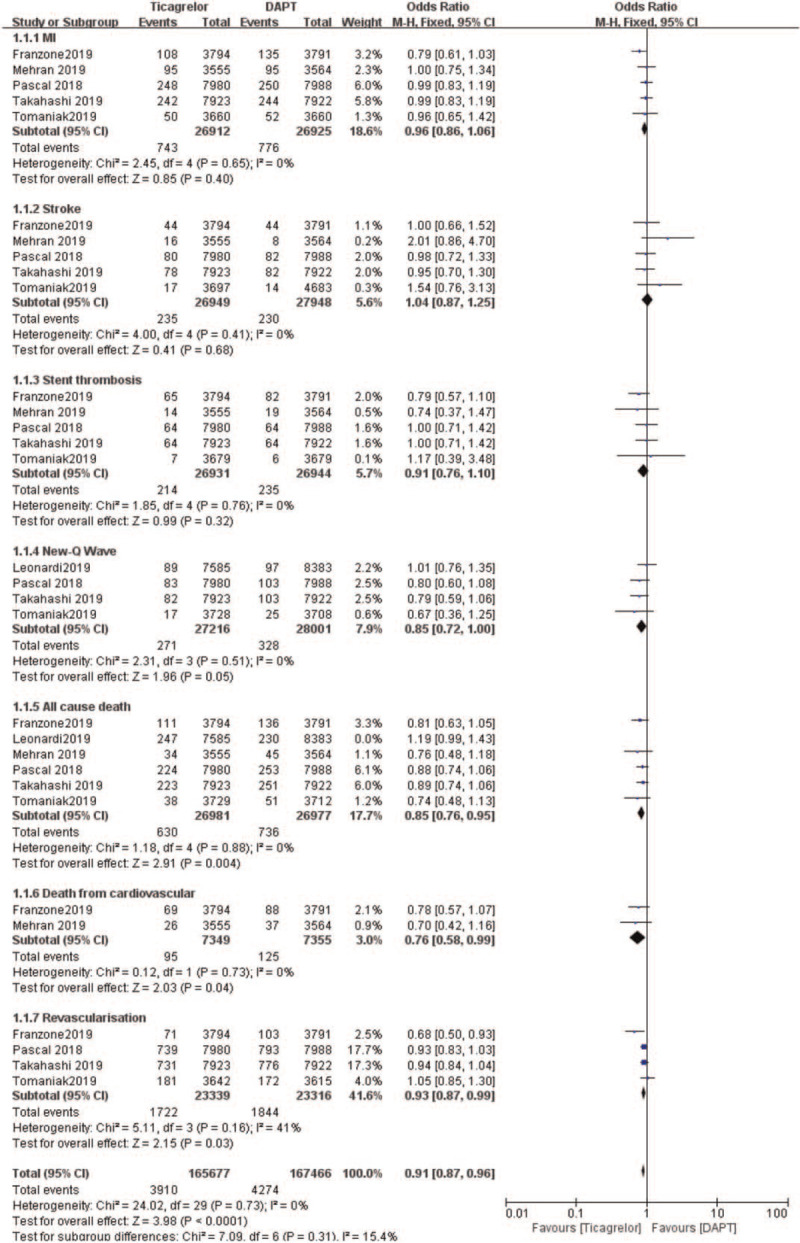
Sensitivity analyses the efficacy (primary outcomes) observed between ticagrelor and DAPT.

The meta-result of “All-cause death”” was tested for heterogeneity (*I*^2^ = 54%, *P* = .05). Sensitivity analysis is used to evaluate the stability and reliability of the combined results of the meta-analysis, by assessing whether the combined results are affected by a single study and have significant changes. By excluding the documents one by one: check whether the heterogeneity has changed after culling one by one. It is found that the heterogeneity has changed after excluding Leonadi et al study, then this article could be the source of the heterogeneity. After removing the article, the results are relatively robust. The sensitivity analysis of 6 documents of this result showed that Leonardi 2019 has a greater impact on heterogeneity. After removing this study, the heterogeneity test show that the remaining 5 documents have no heterogeneity (*I*^2^ = 0%, *P* = .88). After exclusion, fixed-effects model were used for meta-analysis (OR:0.91, 95%CI: 0.87–0.96, *P* < .0001; Fig. 4).

Therefore, this analysis showed that ticagrelor monotherapy was associated with a significantly lower rate of MI, stroke, stent thrombosis, all cause death, death from cardiovascular, and revascularization (OR: 0.91, 95%CI: 0.87–0.96, *P* < .0001) when compared to DAPT.

### 3.5 Secondary outcomes (outcomes representing safety)

Meta-analysis results: heterogeneity test (*P* < .00001, *I*^2^ = 80%), so Random-effects model was used for statistics (OR: 0.80, 95% CI: 0.66, 0.98; *P* = .03), which was statistically significant.

**Figure 5:**
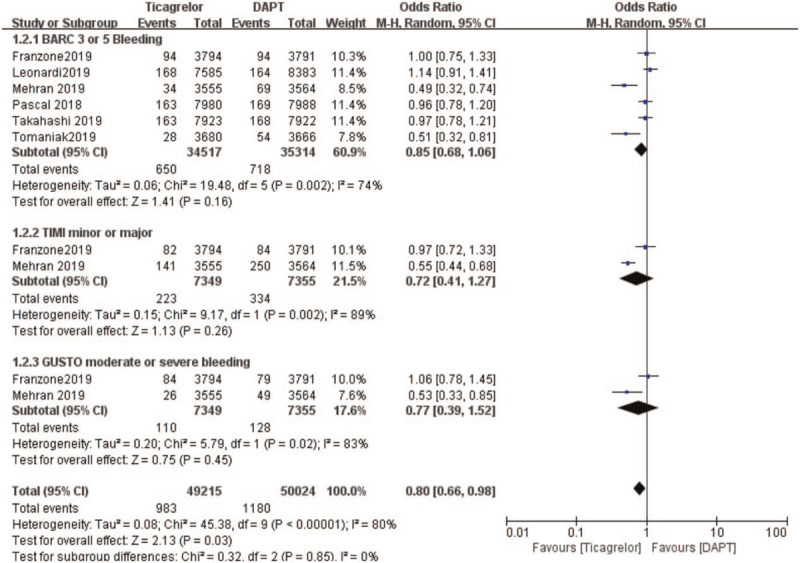
Comparing the bleeding events (secondary outcomes) observed between ticagrelor and DAPT.

This analysis showed that DAPT was associated with a significantly higher rate of BARC3 or 5 bleeding (OR: 0.85, 95% CI: 0.68–1.06; *P* = .16) when compared to ticagrelor. When bleeding was further subdivided, minor or major bleeding was also significantly higher with DAPT (OR: 0.72, 95% CI: 0.41–1.27; *P* = .26). GUSTO moderate or severe bleeding was also significantly higher with DAPT (OR: 0.77, 95% CI: 0.39–1.52; *P* = .45) as shown in Figure 5.

### 3.6 Publication bias

**Figure 6:**
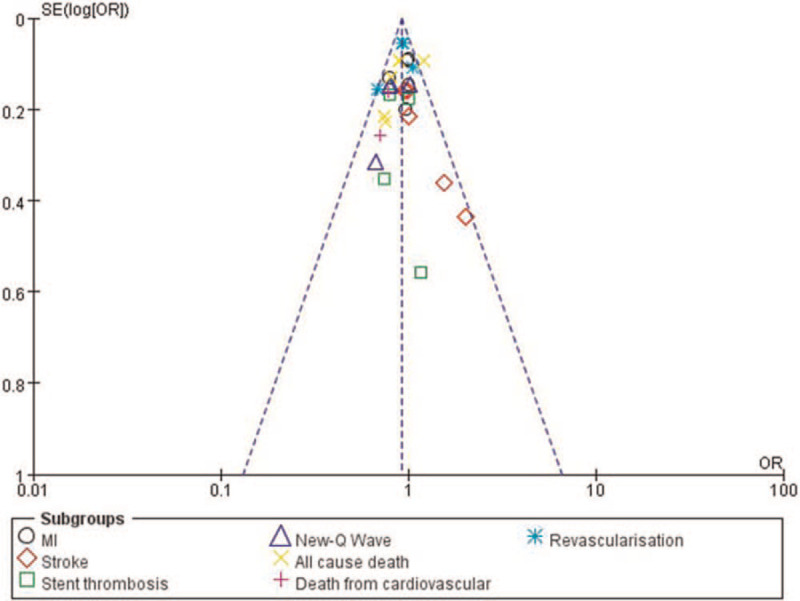
Funnel plot showing publication bias (A).

**Figure 7:**
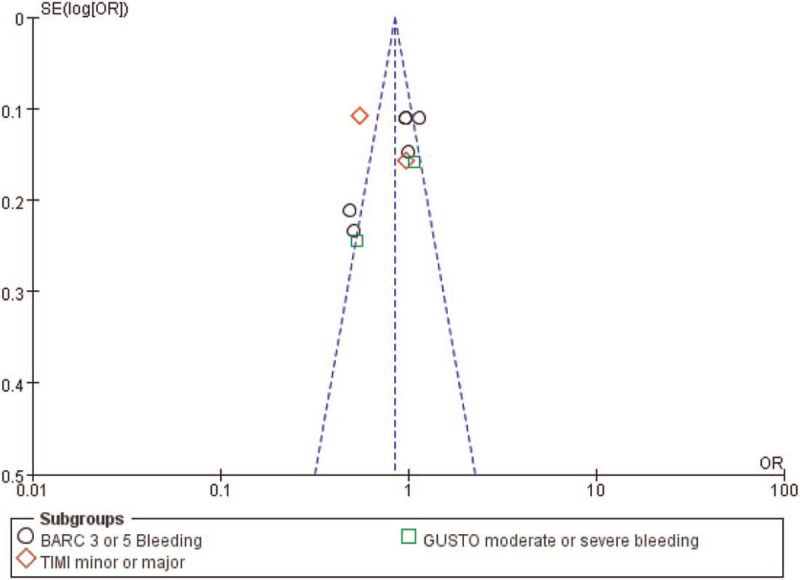
Funnel plot showing publication bias (B).

By observing the funnel plot, there has been a very low evidence of publication bias among the included studies that assessed all the clinical endpoints related to the efficacy and safety observed between ticagrelor and DAPT (Figs. 6 and 7).

## 4 Discussion

According to the current meta-analysis results, ticagrelor monotherapy was associated with a significantly lower rate of MI (OR:0.96, 95%CI: 0.86–1.06, *P* = .40), stroke (OR:1.04, 95%CI: 0.87–1.25, *P* = .68), stent thrombosis (OR:0.91, 95%CI: 0.76–1.10, *P* = .32), all cause death (OR: 0.91, 95%CI: 0.87–0.96, *P* < .0001), death from cardiovascular (OR: 0.76, 95%CI: 0.58–0.99, *P* = .04) and revascularization (OR: 0.93, 95%CI: 0.87–0.99, *P* = .03) when compared to DAPT. DAPT was associated with a significantly higher rate of bleeding events (OR: 0.85, 95% CI: 0.68–1.06; *P* = .16) when compared to ticagrelor monotherapy. Primary outcomes was also significantly higher with DAPT. The question which could most probably be asked at this stage would be about the different mechanisms associated with ticagrelor monotherapy.

The mechanism of ticagrelor is a cyclopentyltriazole pyrimidine type of antiplatelet drug, mainly through selective inhibition of P2 Y12 receptor to achieve the purpose of antiplatelet aggregation, it does not need to be activated by liver metabolism can directly play a role.^[23]^ Therefore, the antiplatelet effect is more obvious and effective, and there is no need to accept genetic testing before antiplatelet intervention.^[24]^ Ticagrelor can significantly reduce all-cause death, MI, and stroke, repeated severe myocardial ischemia, transient ischemic attack, or other arterial thrombosis events, and does not increase the risk of major bleeding. With the extension of the medication time, the benefit of ticagrelor is increasing.^[25]^

Different doses of ticagrelor has also proven to be effective and safe in patients with following PCI. The APOLLO test of Jernberg et al ^[26]^ showed that the incidence of major adverse cardiovascular events within 1 year of MI reached 18.3%. Besides, patients with a history of MI and no events within 1 year after MI have a 20% incidence of hospitalization or death due to MI or stroke within 3 years. The PEGASUS-Post-Myocardial Infarction Thrombolysis 54 study enrolled 21,162 subjects. They were divided into 3 groups at a ratio of 1:1:1 in a randomized, double-blind, and controlled manner, and ticagrelor 90 mg (once a day), ticagrelor 60 mg (twice a day) and placebo. The study suggests that more than 1 year after MI , the ticagrelor group can significantly reduce the risk of cardiovascular death, MI, and stroke, but increase the risk of thrombolysis in myocardial infarction (TIMI) major bleeding.^[24]^

At the same time, compared with the placebo group in terms of safety endpoints and bleeding risk, the extended 60 mg ticagrelor group significantly reduced the risk of the composite endpoint of cardiovascular death, MI, and stroke (7.77% vs 9.04%, *P* = .004),^[27]^ the relative risk is reduced by 16%. Bonaca et al^[28]^ screened patients with low bleeding risk and stratified them according to the number of ischemic risk factors, and found that in ≥2 ischemic risk factor groups, the low-dose ticagrelor group reduced the risk of the primary endpoint The most significant (HR = 0.80, 95%CI = 0.68–0.93, *P* = .0031, absolute risk reduction rate is 1.9%).

In terms of effectiveness and bleeding risk, the standard dose group (ticagrelor 90 mg, 2 times a day) and the low-dose group (ticagrelor 60 mg, 2 times a day) weighed the cardiovascular benefits and the risk of irreversible damage. At the time, the risk ratio was balanced; the 3-year survival rates of the 2 groups were 7.85% and 7.77%, respectively, and the incidence of TIMI major bleeding events in the 2 groups were 2.60% and 2.30% respectively.^[29]^

Studies have found that in low-risk populations, the severity of myocardial damage caused by PCI is usually not sensitive to the level of platelet P2Y12 inhibition.^[30]^ Low-dose ticagrelor used in patients with stable coronary heart disease undergoing elective PCI treatment has a stronger and longer-lasting platelet inhibition rate, and does not significantly affect the absorption of intracellular adenosine and the level of circulating adenosine, nor affect the release of troponin after PCI.

In the same year, the ELECTRA study selected 50 subjects who received PCI treatment after acute myocardial infarction (AMI). Thirty days after AMI, they were randomly divided into groups at a ratio of 1:1 and received downgrade to ticagrelor 60 mg (twice a day) or standard the dose of ticagrelor 90 mg (twice a day) was maintained until 45 days after AMI; the platelet function was measured by the 2 methods of vasodilator-stimulated phosphoprotein test and multielectrode measurement, and the platelet response index of the ticagrelor 60 mg group is higher than the 90 mg ticagrelor group, but still below the threshold of high platelet response, that is, platelet response index >50%; therefore, 1 month after AMI, standardized treatment (ticagrelor 90 mg, daily 2 times) and then reduced to ticagrelor 60 mg (2 times a day) can exert the same platelet inhibitory effect.^[31]^

Ticagrelor has also proven to be effective and safe in patients with type 2 diabetes.^[32]^ Thomas et al^[33]^ conducted a subgroup analysis of type 2 diabetes, and the results suggested that the platelet inhibitory effect before and after the maintenance dose of ticagrelor 60 mg group has nothing to do with type 2 diabetes and whether to use insulin. The pharmacokinetics of Reluo is not affected by the state of diabetes.

The current study on the treatment of degrading in the acute phase aims to evaluate the long-term effect of ticagrelor in the prevention of adverse cardiac events after short-term DAPT. Continuation of ticagrelor for 15 months after the DAPT after PCI significantly reduces the risk of bleeding events and does not increase ischemic events. Both studies were explored and analyzed in terms of shortening the duration of the DAPT. At present, there is no evidence-based basis for reducing the dose of DAPT in patients with AMI or stable coronary heart disease within 1 year, and most of the existing studies are singlecenter clinical trials, and larger sample sizes and larger multicenters are needed. Clinical research provides evidence-based evidence to seek a balance between efficacy and risk for individualized antiplatelet therapy.

There is currently some controversy about the relationship between DAPT time and prognosis after PCI. Some study found that Long-term DAPT was associated with increased risk for major bleeding.^[34]^ Kim et al^[35]^ found that among patients with acute coronary syndromes treated with drug-eluting stents, ticagrelor monotherapy after 3 months of dual antiplatelet therapy, compared with ticagrelor-based 12-month dual antiplatelet therapy, resulted in a modest but statistically significant reduction in a composite outcome of major bleeding and cardiovascular events at 1 year. Yang et al^[36]^ found that after 6 months, the DAPT was changed to ticagrelor monotherapy to be safe and feasible for 1 year. It not only effectively inhibits platelet aggregation, but also does not increase the incidence of adverse cardiovascular events; at the same time, its bleeding events are less than that of the DAPT at 1 year (4.44% vs 8.89%). These research is consistent with our meta-analysis results.

## 5 Limitations

First of all, due to only 6 studies were included in meta-analyisis, the results of this analysis might have been affected. In addition, the country of patients’ enrollment is not clarified. Another limitation of this analysis could be some study primary and secondary outcomes were few. Not having included such an important information might contribute to the limitation of this research. Finally, this research still need to use multicenter, large sample RCT for further verification. Fortunately, the total sample size of the 6 articles included this time is large.

## 6 Conclusion

Ticagrelor monotherapy after short-term DAPTmay optimize ischemic and bleeding risks. This analysis showed that ticagrelor monotherapy was associated with a significantly lower rate of MI, stroke, stent thrombosis, all cause death, death from cardiovascular, and revascularization when compared to DAPT.

However, because antiplatelet drug treatment is affected by many factors such as the patient's age, CRUSADE score, medication compliance, and other factors, the formulation of antiplatelet regimens after PCI needs to be determined according to the specific conditions of the patient.

In the practical application, usage of ticagrelor alone. It was found to be sufficient to protect the patients and to have a low bleeding incidence compared to dual therapy. Using ticagrelor alone as early as possible can also reduce the patient's cost burden.

## Acknowledgments

We would like to thank Professor Chuan-hua Yang (Cardiovascular Division, Dean of the Affiliated Hospital of Shandong University of Traditional Chinese Medicine) for supervising the statistical analyses. We would like to thank Yong Wang MD for assistance with editing (limited to collation of author comments and formatting for submission). We also thank all the participant who were provided suggestions of this study.

## Author contributions

**Conceptualization:** Wen-bin Zhang, Zhen Wang.

**Data curation:** Wen-bin Zhang, Zhen Wang, Li-nan Liu, Yang Liu.

**Formal analysis:** Wen-bin Zhang, Li-nan Liu.

**Investigation:** Wen-bin Zhang, Li-nan Liu, Yang Liu.

**Methodology:** Wen-bin Zhang, Li-nan Liu, Yang Liu.

**Project administration:** Zhen Wang.

**Resources:** Wen-bin Zhang, Yang Liu.

**Software:** Wen-bin Zhang, Zhen Wang, Yang Liu.

**Supervision:** Zhen Wang.

**Validation:** Zhen Wang.

**Visualization:** Zhen Wang.

**Writing – original draft:** Wen-bin Zhang.

**Writing – review & editing:** Zhen Wang.
